# The future objectives of the International Mouse Phenotyping Consortium (IMPC): functional evaluation of human disease-relevant cis-regulatory elements in the mouse genome

**DOI:** 10.1007/s00335-026-10258-9

**Published:** 2026-07-24

**Authors:** Naoki Kubo, Akiko Oguchi, Hiroshi Masuya, Takanori Amano, Akihiko Sakashita, Hideya Kawaji, Takeya Kasukawa, Shinya Oki, Shinya Ayabe, Toyoyuki Takada, Atsuo Ogura, Kimiko Inoue, Yasuhiro Murakawa, Masaru Tamura, Atsushi Yoshiki, Toshihiko Shiroishi

**Affiliations:** 1 BioResource Research Center (BRC), RIKEN, Tsukuba, Japan; 2 Laboratory for Gene Structure and Regulation, Center for Integrative Medical Sciences (IMS), RIKEN, Yokohama, Japan; 3 Research Center for Genome & Medical Sciences, Tokyo Metropolitan Institute of Medical Science, Tokyo, Japan; 4 Laboratory for Large-Scale Biomedical Data Technology, Center for Integrative Medical Sciences (IMS), RIKEN, Yokohama, Japan; 5 Institute of Resource Development and Analysis, Kumamoto University, Kumamoto, Japan; 6 Institute for the Advanced Study of Human Biology, Kyoto University, Kyoto, Japan; 7 Department of Pathology and Biology of Diseases, Graduate School of Medicine, Kyoto University, Kyoto, Japan

## Abstract

The International Mouse Phenotyping Consortium (IMPC) has established a large-scale functional genomics resource by systematically generating and phenotyping knockout mouse lines, linking gene function to mammalian phenotypes. However, interpreting disease-associated non-coding variants remains particularly challenging due to their abundance, context-dependent activity, and the complexity of gene regulation. Genome-wide association studies (GWAS) have shown that many disease-associated loci map to non-coding regions. In addition, recent large-scale consortia have catalogued millions of candidate cis-regulatory elements (CREs) across mammalian genomes; however, predicting which elements contribute to disease-relevant gene regulation and organismal phenotypes remains difficult. Together, these observations highlight disease-relevant CREs as an important but still underexplored component of human disease mechanisms. In this white paper, we outline strategies to address this challenge: (i) prioritization of disease-relevant candidate CREs, (ii) genome editing in mice to functionally evaluate CREs, and (iii) the establishment of interdisciplinary working groups. By extending its activities beyond protein-coding sequences, the IMPC has a unique opportunity to define the functional and phenotypic impact of disease-relevant CREs in vivo at scale, thereby improving our understanding of how non-coding regulatory elements contribute to mammalian phenotypes and human disease.

## Introduction

Human genetics has increasingly highlighted the importance of non-coding variation in human disease. While many disease-causing variants have been identified within protein-coding sequences, genome-wide association studies (GWAS) have shown that a substantial proportion of disease-associated loci map to non-coding regions of the genome (Khurana et al. 2016; Perenthaler et al. 2019; Rheinbay et al. 2020). At the same time, large-scale epigenomic consortia, including ENCODE and FANTOM, have identified millions of candidate functional elements across human and mouse non-coding genomes (Consortium 2012; Consortium et al. 2014, 2020). These findings suggest that *cis*-regulatory elements (CREs), including promoters, enhancers, silencers, and insulators, play important roles in gene regulation, disease susceptibility, and pathogenesis. Among these classes of CREs, the present proposal primarily focuses on enhancers because many disease-associated loci are thought to exert their effects through enhancer-mediated gene regulation. However, despite the growing recognition of their importance, the functional consequences of most disease-associated CREs remain poorly understood.

The International Mouse Phenotyping Consortium (IMPC) has delivered an unprecedented, systematic effort to generate and phenotype knockout moues lines for a large fraction of protein-coding genes, establishing a foundational resource for linking gene function to mammalian biology and human disease (Birling et al. 2021; Dickinson et al. 2016; Meehan et al. 2017; Wilson et al. 2026). Since its inception, the IMPC has produced and comprehensively analyzed knockout mouse lines for approximately 10,000 genes, uncovering over 100,000 statistically robust genotype–phenotype associations across diverse biological systems. These high-throughput standardized datasets are made publicly available, enabling genome-wide annotation of gene function and facilitating cross-species interpretation relevant to human disease. Collectively, the IMPC has generated one of the most comprehensive functional genomics resources currently available. Despite this remarkable progress, the functional and phenotypic consequences of non-coding regulatory elements remain much less well characterized (Lloyd et al. 2020). Recent large-scale consortium studies in epigenomics have annotated millions of cell type-specific candidate CREs, including enhancers, across the human genome and other mammalian species. However, their abundance makes it difficult to predict which elements have functional effects on gene regulation and disease-relevant phenotypes. Moreover, because a single CRE can regulate multiple genes or modulate gene expression in a graded manner rather than a simple on–off state, perturbation of CREs may generate phenotypes distinct from those observed in conventional coding gene knockouts. Consequently, systematic functional evaluation of disease-relevant CREs represents an important next step toward understanding how non-coding variation contributes to human disease.

Recent advances in epigenomics, genome editing, and computational technologies provide new opportunities to prioritize candidate CREs and evaluate their functional roles through mouse phenotyping. In this white paper, we outline possible strategies that the IMPC could adopt for selecting disease-relevant target CREs (Sections I), genome editing in mice (Section II), and establishing interdisciplinary working groups (Section III) to better understand how non-coding regulatory elements contribute to mammalian phenotypes and the molecular mechanisms underlying human disease.

### Selection of target CREs for developing mouse models

**Fig. 1:**
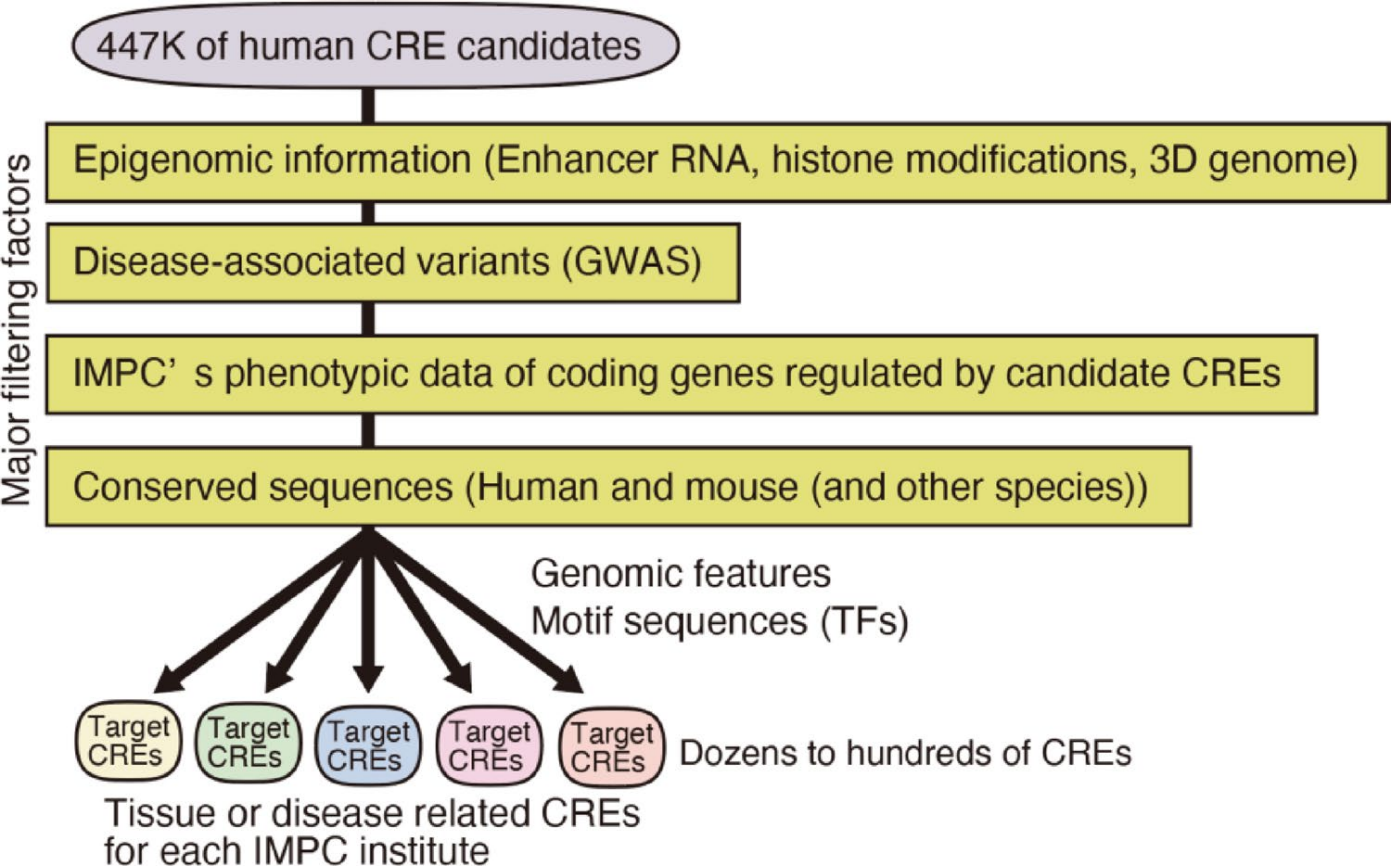
A strategy for selecting target CREs for generating mouse models

Although non-coding sequences constitute 98% of the genome, the proportion that directly contributes to phenotypes appears relatively small, and the molecular mechanisms underlying their abnormal phenotypes are not fully understood (Khurana et al. 2016; Panigrahi and O’Malley 2021; Perenthaler et al. 2019; Rheinbay et al. 2020). However, recent advances in genome and epigenome research, together with computational approaches, are now providing new opportunities to identify candidate CREs for functional investigation (Consortium 2012; Cusanovich et al. 2018; Gorkin et al. 2020; Oguchi et al. 2024; Zhang et al. 2021). Below, we describe several potential approaches for prioritizing target CREs for genome editing in mice within the framework of the IMPC (Fig. 1).

### Cell type-specific epigenomic and enhancer RNA data

To address the functional role of non-coding sequences, a variety of epigenomic information, including open chromatin (ATAC-seq), DNA and histone modifications (WGBS, ChIP-seq, CUT&RUN) as well as 3D genome information (Hi-C, Micro-C, and HiChIP (PLAC-seq), is widely used for target CREs identification (Chen et al. 2016; Consortium et al. 2020; Luo et al. 2020; Schmitt et al. 2016). For example, ATAC-seq peaks and histone H3K27ac and H3K4me1 marks are generally used for predicting active enhancers, and their peak levels and chromatin contacts with gene promoters would help determine genome editing design and prioritize targets. Furthermore, recent study reported that improved detection of enhancer RNA (eRNA) from distal elements would be useful for identifying candidate CREs that regulate gene activation (Hirabayashi et al. 2019; Oguchi et al. 2024). Importantly, compared to ATAC-seq signals, which detect a wide range of distal elements, including those not associated with active enhancers, these eRNA signals provide a more precise representation of active enhancer regions and are highly enriched at disease-related SNPs. Meanwhile, since many genes are surrounded by multiple active enhancers, it is challenging to determine which one is crucial for activating the target gene or whether each distal element is equally important. Therefore, 3D genome information, particularly data on enhancer-promoter chromatin contacts and genomic features would be helpful for prioritizing the target CREs to be mutated in mice　(Kubo et al. 2021, 2024; Oguchi et al. 2024). The genome editing strategy in such cases is also discussed in Section II below. Public resources generated by large-scale consortia, including ENCODE, FANTOM, and the 4D Nucleome project, together with integrated databases such as ChIP-Atlas and LooP Catalog, provide valuable resources for assessing the confidence of candidate CREs (Consortium 2012; Consortium et al. 2014; Dekker et al. 2017; Oki et al. 2018; Reyna et al. 2025). Since public datasets for these analyses are still limited in some tissues and cell types, one practical approach would be to begin with cell types and diseases for which relevant datasets are already available, while incorporating newly generated datasets when necessary to further refine candidate selection.

### Association with known diseases and phenotypes

Since each research center participating in the IMPC has its own interest and expertise in specific diseases and phenotypes, the target CREs could be prioritized based on the specialized research focus of each institute. Utilizing GWAS datasets to narrow down candidate CREs associated with particular diseases would be a valuable approach. Additionally, if each research institute focuses on specific tissues or cell types, lineage-specific genes can be listed using publicly available bulk or single-cell transcriptomic datasets. Importantly, IMPC has already identified thousands of coding genes whose disruption cause severe phenotypes, including embryonic lethality (Birling et al. 2021; Dickinson et al. 2016). If disruption of a coding gene causes a severe phenotype, perturbation of CREs associated with that gene may have a higher likelihood of producing detectable phenotypic consequences. Therefore, prioritizing CREs associated with genes that cause severe phenotypes upon disruption would be an effective strategy to increase the likelihood of identifying disease-relevant phenotypes. Indeed, the IMPC has reported that the knockouts in approximately 2,000 coding genes cause embryonic or pre-weaning lethality. If deletions of CREs that regulate such essential genes are viable and show phenotypes during adolescence and adulthood, they may uncover disease-relevant phenotypes that cannot be studied in conventional coding gene knockout models due to early lethality. Such models could therefore provide important insights into regulatory mechanisms underlying human diseases. In addition, prioritizing disease-relevant CREs associated with genes implicated in human diseases would not only increase the likelihood of detecting phenotypic consequences but also facilitate interpretation of the resulting phenotypes. While the present strategy primarily focuses on disease-relevant CREs identified through GWAS, the framework can be readily extended to prioritize rare disease-associated non-coding variants from disease-specific sequencing studies when expanding the range of target diseases.

### Evolutionary sequence conservation

To evaluate the translatability and evolutionary conservation of human disease-associated CREs to mouse model, sequence conservation between human and mouse serves as a basic criterion for selecting target CREs. In the fanta.bio (v1.1.0) database (https://fanta.bio) (Nobusada et al. 2025), approximately 447,000 CREs have been identified in the human genome by CAGE (Cap Analysis of Gene Expression) analysis (Consortium et al. 2014; Kodzius et al. 2006). Among these, around 210,000 CREs can be mapped to the mouse genome using the liftOver tool (Hinrichs et al. 2006), with more than 80% base matches. Of these, around 80,000 regions have been identified as CREs by CAGE analysis in mice, and about 30,000 of these CREs exhibit more than 95% sequence homology with the humans. These results suggest that sequence conservation can serve as a crucial filter to narrow down the number of candidates. Given these sequence-based phylogenetic approaches, a machine learning-based prediction methods, such as Tissue-Aware Conservation Inference Toolkit (TACIT) (Kaplow et al. 2023), could also be useful for inferring cross-species differences in enhancer activity from nucleotide sequence variation.

### Transcription factor interactions

If further narrowing down of CREs targets is required, it is reasonable to prioritize based on the transcription factors (TFs) that bind to the CREs. For instance, if null mutations of TFs cause phenotypes in the disease-relevant tissues, CREs that interact with those TFs may also contribute to those phenotypes. The ChIP-Atlas database (https://chip-atlas.org/), linked from the fanta.bio database, compiles TF binding sites derived from all publicly available ChIP-seq data (Abugessaisa et al. 2021; Oki et al. 2018). The information of the TF binding profiles is important to evaluate CREs as potential targets for the knockout mouse production.

Lastly, if CRE knockout mice do not exhibit any observable phenotypes despite rigorous multilayer filtering of candidate CREs as described above, such findings would still provide important insights into the robustness and redundancy of gene regulatory mechanisms and improve our understanding of disease-relevant regulatory architecture.

### A case study of CRE selection in CD4^+^ T cell

**Fig. 2:**
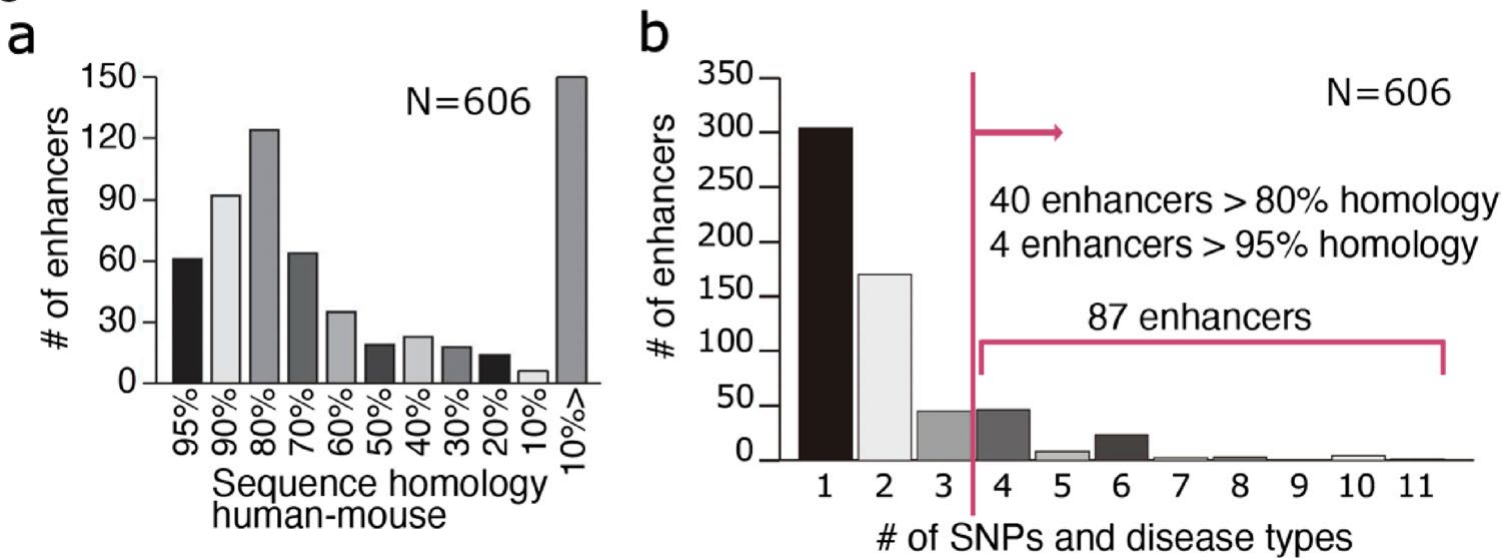
**a** A bar graph showing the number of candidate enhancers with eRNA expression in CD4+ T cell and at least one GWAS-SNP associated with immune-mediated diseases, classified based on sequence homology between human and mouse. **b** A bar graph showing the number of candidate enhancers classified based on the number of SNPs and disease types of immune-mediated diseases

Based on the multilayer filtering strategy for target CREs described above, we utilized datasets of human genomic and epigenomic information, including eRNA data from CD4 + T cell and GWAS-SNPs associated with immune-mediated diseases. In our previous study, we identified 606 candidate enhancers using these eRNA and GWAS datasets (Oguchi et al. 2024). Among these, 277 and 61 candidate enhancers were found to have at least 80% and 95% sequence homology with the mouse genome, respectively. We then hypothesized that the overlap of multiple GWAS-SNPs and disease types with the candidate enhancers would contribute to a broader phenotype of CRE mutations. By taking the number of GWAS-SNPs identified in each disease into account, further narrowing down could be achieved (Fig. 2). Finally, for instance, 40 enhancers with 80% sequence homology and 4 enhancers with 95% sequence homology were associated with more than 4 GWAS-SNPs and disease types. Among them, one CRE has indeed been reported carrying a mutation that results in a marked phenotype (Sokhi et al. 2018). A few dozen candidates per tissue or disease group would be considered a reasonable number for each IMPC center to handle.

## Strategies to enhance the efficacy of genome editing for deleting CREs in mice

Because both loss-of-function and gain-of-function variants can contribute to disease etiology, alternative approaches such as variant knock-in or base editing may be appropriate for selected loci. However, deletion-based perturbation represents the most practical and standardized first-line strategy for large-scale functional evaluation of CREs within the scope of the present proposal, consistent with current IMPC methodologies.

**Fig. 3:**
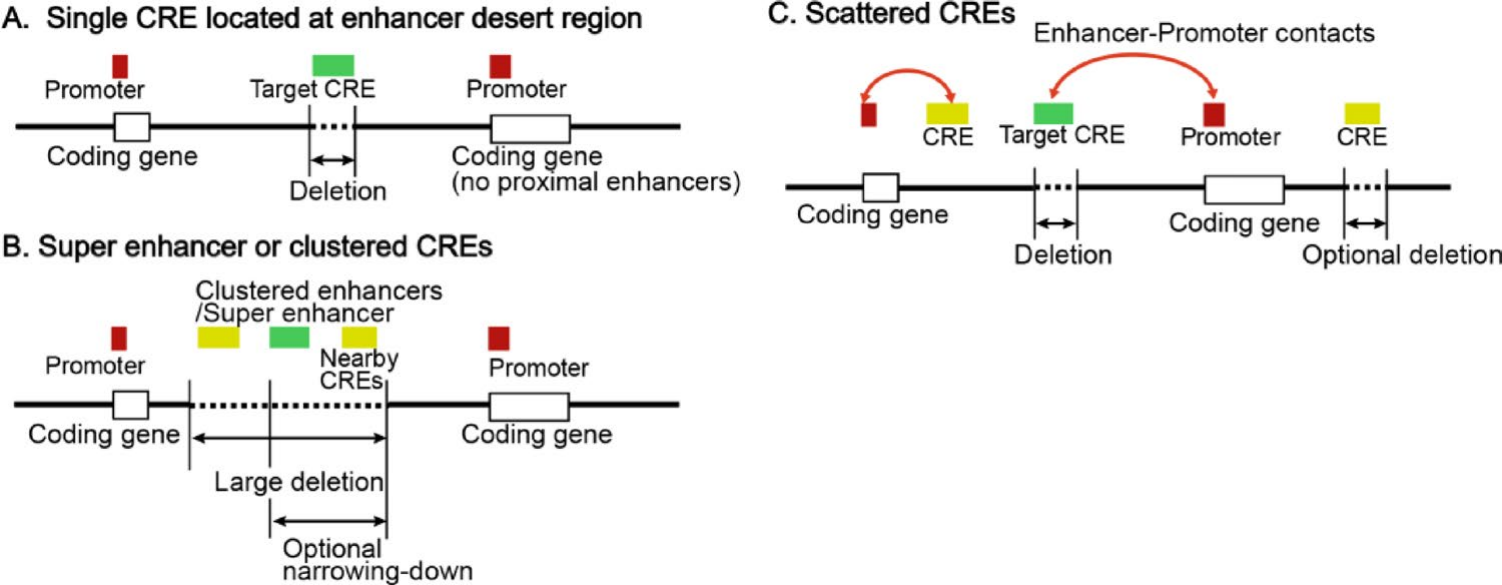
Strategies for deleting target CREs in mouse genome

Despite employing the multilayer filtering approaches described above, there remains a possibility that the deletion of a single CRE may not result in an observable phenotype due to functional redundancy provided by multiple nearby enhancers (Kubo et al. 2021; Kvon et al. 2021; Osterwalder et al. 2018). Therefore, it is important to develop strategies to enhance the likelihood of observing a phenotype, which in turn ensures that the absence of a phenotype itself becomes a scientifically meaningful finding. Outlined below are several stepwise condition-based approaches for deleting target CREs in mice (Fig. 3).

### Deletion of a single CRE located at enhancer desert region

If the target CRE is located alone without any enhancers nearby, the transcriptional regulation of the target gene is likely to depend on its enhancer activity. Therefore, deleting it alone would be a reasonable approach. Given that mutations in promoter regions often result in effects equivalent to coding null mutations, we would exclude them as target CREs in this proposal.

### Large deletion for super enhancer or clustered CREs

Among cases where the target CRE is located near multiple enhancers, there are instances where a subset of them forms cluster known as super-enhancer. In such cases, it may be worth considering the broad deletion of these regions as a single unit. After obtaining phenotypes caused by the large deletions, further validation by narrowing down the number of deleted CREs might be conducted. If the deletion of such large regions or highly active super-enhancers does not result in any observable phenotypes, it could still raise scientifically important questions and be reported as a meaningful finding.

### Deletion of scattered CREs simultaneously

If the conditions mentioned above are not met, this would mean that several active enhancers are scattered around the target CRE without forming a cluster. In such cases, unless there is clear evidence of interaction of the CRE and promoter, as indicated by strong enhancer-promoter contacts, it would be advisable to either avoid creating model mice or, after weighing the effort required for mouse model generation, proceed with creating models in which those multiple candidate enhancers are deleted simultaneously.

Besides localized non-coding regions, genomic alterations on a megabase scale—including those impacting topologically associated domains (TADs) or chromosome-level structures—have also been implicated in disease and phenotypic outcomes (He et al. 2014; Lupianez et al. 2015). Chromosome-level genome editing technologies such as CRISMERE would be useful in such cases (Schaeffer et al. 2023).

### Systematic selection of CREs to delete using AI-based prediction tools

Recent advances in machine learning tools in genomics and epigenomics have been remarkable, and such technologies are expected to play an important role in functional genomics (Avsec et al. 2026; Brixi et al. 2026). For example, AlphaGenome (Avsec et al. 2026), which has been trained on large-scale mouse and human epigenomic datasets, supports variant-effect prediction and in silico mutagenesis within genomic contexts of up to ~ 1 Mb. Although accurate prediction of the effects of perturbing distal enhancer regulation in certain mouse cell types will likely require further improvement, such tools will enable estimation of the effect sizes of variants on phenotypic outcomes and facilitate the design of high-confidence CRE deletions.

Furthermore, the large-scale CRE perturbation and phenotyping datasets generated through this effort would provide a unique training resource for AI-based phenotype prediction. These datasets would provide a foundation for models capable of linking non-coding genomic variation to organismal phenotypes. The systematic generation of CRE–phenotype datasets may therefore contribute to future phenotype prediction beyond current models of genome regulation.

## Enhancement of phenotypic analysis through international collaboration by IMPC

Advancing to non-coding sequences as the next target for the IMPC, which has thus far focused on protein-coding genes, is an inevitable step in the progression of phenotypic research. Although additional analyses and strategies will be required to select target CREs for genome editing, as described above, the IMPC has a significant advantage in utilizing comprehensive phenotypic data from knockout mice for coding genes as a valuable reference for assessing the effects of CRE mutations in non-coding sequences. Since CRE activity is tissue-specific and its effect on target gene is variable, phenotypic analysis at the whole-body level, along with comparison to the effects of coding gene deletion, would provide more comprehensive information. Meanwhile, even if no observable phenotype is detected, demonstrating that highly active and conserved CREs have no impact on in vivo phenotypes in some cases could provide meaningful insights into mammalian genome function. In practice, when assigning tissue- and disease-specific target CREs to each research center within the IMPC, there may be cases where overlapping enhancers are shared across multiple tissues and disease groups. Therefore, a centralized working group should be established within the IMPC to ensure transparent allocation of target CREs. This group could maintain a shared registry of candidate CREs, assigned research centers, and project status. When multiple centers are interested in the same CRE or disease area, target assignment could be coordinated based on each center’s expertise and available phenotyping platforms. Such a framework would help avoid unnecessary duplication while leveraging overlapping interests for collaborative work.

In addition to organismal phenotyping, transcriptomic profiling should be considered a key component of functional studies of CREs. Systematic gene expression analyses, including bulk and single-cell transcriptomic approaches, would provide valuable information regarding tissue- and cell type-specific effects of CRE perturbation on neighboring genes and regulatory networks. Such molecular profiling could also serve as an efficient intermediate screening step. For example, transcriptomic analyses of founder mice may help identify CRE perturbations with measurable regulatory consequences and guide decisions regarding subsequent large-scale mouse production and comprehensive phenotyping efforts. Beyond target selection, integrated epigenomic and transcriptomic analyses of affected tissues in CRE mutant mice will provide deeper insights into the underlying pathogenic mechanisms. Such analyses will be particularly important when the target CRE functions as a regulatory hub that could potentially affect multiple genes, or when CRE perturbation induces graded changes in gene expression that give rise to phenotypes not observed in conventional gene KO models.

Therefore, to effectively implement this strategy for the functional evaluation of disease-relevant CREs, coordinated efforts across the IMPC and related communities will be required. A working group comprising researchers from genetics, genomics, epigenomics, and bioinformatics would facilitate the prioritization of tissue- and disease-specific target CREs and the integration of downstream molecular analyses in phenotyped mouse models. While new approach methodologies (NAMs), including organoids, organs-on-chip, and computational prediction models, are expected to play increasingly important roles in functional genomics, many disease-relevant phenotypes—including inter-organ communication, metabolism, immune responses, and behavior—cannot yet be fully recapitulated outside the context of a whole organism. Therefore, mouse phenotyping will remain an essential component of functional evaluation of disease-relevant CREs. At the same time, data generated through IMPC studies would provide a unique resource linking perturbations of non-coding regulatory elements to organismal phenotypes, a critical type of training data that is currently lacking. Such datasets could help validate and improve future computational and experimental models, thereby contributing to long-term efforts to reduce animal use in accordance with the 3Rs principles. Ultimately, these coordinated activities would support the development of a community-wide resource for understanding how disease-relevant CREs contribute to gene regulation, mammalian phenotypes, and human disease mechanisms. Such collaborative efforts would be essential for the next stage of IMPC activities, particularly in expanding its impact from protein-coding genes to disease-relevant regulatory elements within the non-coding genome.

## Data Availability

No datasets were generated or analysed during the current study.

## References

[CR1] Abugessaisa I, Ramilowski JA, Lizio M, Severin J, Hasegawa A, Harshbarger J, Kondo A, Noguchi S, Yip CW, Ooi JLC, Tagami M, Hori F, Agrawal S, Hon CC, Cardon M, Ikeda S, Ono H, Bono H, Kato M, Hashimoto K, Bonetti A, Kato M, Kobayashi N, Shin J, de Hoon M, Hayashizaki Y, Carninci P, Kawaji H, Kasukawa T (2021) FANTOM enters 20th year: expansion of transcriptomic atlases and functional annotation of non-coding RNAs. Nucleic Acids Res 49:D892–D898 33211864 10.1093/nar/gkaa1054 PMC7779024

[CR2] Avsec Z, Latysheva N, Cheng J, Novati G, Taylor KR, Ward T, Bycroft C, Nicolaisen L, Arvaniti E, Pan J, Thomas R, Dutordoir V, Perino M, De S, Karollus A, Gayoso A, Sargeant T, Mottram A, Wong LH, Drotar P, Kosiorek A, Senior A, Tanburn R, Applebaum T, Basu S, Hassabis D, Kohli P (2026) Advancing regulatory variant effect prediction with AlphaGenome. Nature 649:1206–1218 41606153 10.1038/s41586-025-10014-0 PMC12851941

[CR3] Birling MC, Yoshiki A, Adams DJ, Ayabe S, Beaudet AL, Bottomley J, Bradley A, Brown SDM, Burger A, Bushell W, Chiani F, Chin HG, Christou S, Codner GF, DeMayo FJ, Dickinson ME, Doe B, Donahue LR, Fray MD, Gambadoro A, Gao X, Gertsenstein M, Gomez-Segura A, Goodwin LO, Heaney JD, Herault Y, de Angelis MH, Jiang ST, Justice MJ, Kasparek P, King RE, Kuhn R, Lee H, Lee YJ, Liu Z, Lloyd KCK, Lorenzo I, Mallon AM, McKerlie C, Meehan TF, Fuentes VM, Newman S, Nutter LMJ, Oh GT, Pavlovic G, Ramirez-Solis R, Rosen B, Ryder EJ, Santos LA, Schick J, Seavitt JR, Sedlacek R, Seisenberger C, Seong JK, Skarnes WC, Sorg T, Steel KP, Tamura M, Tocchini-Valentini GP, Wang CL, Wardle-Jones H, Wattenhofer-Donze M, Wells S, Wiles MV, Willis BJ, Wood JA, Wurst W, Xu Y, International Mouse Phenotyping C, Teboul L, Murray SA (2021) A resource of targeted mutant mouse lines for 5,061 genes. Nat Genet 53, 416–419 10.1038/s41588-021-00825-y PMC8397259 33833456

[CR4] Brixi G, Durrant MG, Ku J, Naghipourfar M, Poli M, Sun G, Brockman G, Chang D, Fanton A, Gonzalez GA, King SH, Li DB, Merchant AT, Nguyen E, Ricci-Tam C, Romero DW, Schmok JC, Taghibakhshi A, Vorontsov A, Yang B, Deng M, Gorton L, Nguyen N, Wang NK, Pearce MT, Simon E, Adams E, Amador ZJ, Ashley EA, Baccus SA, Dai H, Dillmann S, Ermon S, Guo D, Herschl MH, Ilango R, Janik K, Lu AX, Mehta R, Mofrad MRK, Ng MY, Pannu J, Re C, St John J, Sullivan J, Tey J, Viggiano B, Zhu K, Zynda G, Balsam D, Collison P, Costa AB, Hernandez-Boussard T, Ho E, Liu MY, McGrath T, Powell K, Pinglay P, Burke DP, Goodarzi H, Hsu PD, Hie BL (2026) Genome modelling and design across all domains of life with Evo 2. Nature. 10.1038/s41586-026-10176-5 41781614 10.1038/s41586-026-10176-5 PMC13128491

[CR5] Chen L, Ge B, Casale FP, Vasquez L, Kwan T, Garrido-Martin D, Watt S, Yan Y, Kundu K, Ecker S, Datta A, Richardson D, Burden F, Mead D, Mann AL, Fernandez JM, Rowlston S, Wilder SP, Farrow S, Shao X, Lambourne JJ, Redensek A, Albers CA, Amstislavskiy V, Ashford S, Berentsen K, Bomba L, Bourque G, Bujold D, Busche S, Caron M, Chen SH, Cheung W, Delaneau O, Dermitzakis ET, Elding H, Colgiu I, Bagger FO, Flicek P, Habibi E, Iotchkova V, Janssen-Megens E, Kim B, Lehrach H, Lowy E, Mandoli A, Matarese F, Maurano MT, Morris JA, Pancaldi V, Pourfarzad F, Rehnstrom K, Rendon A, Risch T, Sharifi N, Simon MM, Sultan M, Valencia A, Walter K, Wang SY, Frontini M, Antonarakis SE, Clarke L, Yaspo ML, Beck S, Guigo R, Rico D, Martens JHA, Ouwehand WH, Kuijpers TW, Paul DS, Stunnenberg HG, Stegle O, Downes K, Pastinen T, Soranzo N (2016) Genetic Drivers of Epigenetic and Transcriptional Variation in Human Immune Cells. Cell 167:1398–1414 e1324 27863251 10.1016/j.cell.2016.10.026 PMC5119954

[CR6] Consortium EP (2012) An integrated encyclopedia of DNA elements in the human genome. Nature 489:57–74 22955616 10.1038/nature11247 PMC3439153

[CR7] Consortium F, the RP, Clst, Forrest AR, Kawaji H, Rehli M, Baillie JK, de Hoon MJ,Haberle V, Lassmann T, Kulakovskiy IV, Lizio M, Itoh M, Andersson R, Mungall CJ, Meehan TF, Schmeier S, Bertin N, Jorgensen M, Dimont E, Arner E, Schmidl C, Schaefer U, Medvedeva YA, Plessy C, Vitezic M, Severin J, Semple C, Ishizu Y, Young RS, Francescatto M,Alam I, Albanese D, Altschuler GM, Arakawa T, Archer JA, Arner P, Babina M, Rennie S, Balwierz PJ, Beckhouse AG, Pradhan-Bhatt S, Blake JA, Blumenthal A, Bodega B, Bonetti A, Briggs J, Brombacher F, Burroughs AM, Califano A, Cannistraci CV, Carbajo D, Chen Y, Chierici M, Ciani Y, Clevers HC, Dalla E, Davis CA, Detmar M, Diehl AD, Dohi T,Drablos F, Edge AS, Edinger M, Ekwall K, Endoh M, Enomoto H, Fagiolini M, Fairbairn L, Fang H, Farach-Carson MC, Faulkner GJ, Favorov AV, Fisher ME, Frith MC, Fujita R, Fukuda S, Furlanello C, Furino M, Furusawa J, Geijtenbeek TB, Gibson AP, Gingeras T, Goldowitz D, Gough J, Guhl S, Guler R, Gustincich S, Ha TJ, Hamaguchi M, Hara M,Harbers M, Harshbarger J, Hasegawa A, Hasegawa Y, Hashimoto T, Herlyn M, Hitchens KJ, Ho Sui SJ, Hofmann OM, Hoof I, Hori F, Huminiecki L, Iida K, Ikawa T, Jankovic BR, Jia H, Joshi A, Jurman G, Kaczkowski B, Kai C, Kaida K, Kaiho A, Kajiyama K, Kanamori-Katayama M, Kasianov AS, Kasukawa T, Katayama S, Kato S, Kawaguchi S, Kawamoto H, Kawamura YI, Kawashima T, Kempfle JS, Kenna TJ, Kere J, Khachigian LM, Kitamura T, Klinken SP, Knox AJ, Kojima M, Kojima S, Kondo N, Koseki H, Koyasu S, Krampitz S, Kubosaki A, Kwon AT, Laros JF, Lee W, Lennartsson A, Li K, Lilje B, Lipovich L, Mackay-Sim A, Manabe R, Mar JC, Marchand B, Mathelier A, Mejhert N, Meynert A, Mizuno Y, de Lima Morais DA, Morikawa H, Morimoto M, Moro K, Motakis E, Motohashi H, Mummery CL, Murata M, Nagao-Sato S, Nakachi Y, Nakahara F, Nakamura T, Nakamura Y, Nakazato K, van Nimwegen E, Ninomiya N, Nishiyori H, Noma S, Noma S, Noazaki T, Ogishima S, Ohkura N, Ohimiya H, Ohno H, Ohshima M, Okada-Hatakeyama M, Okazaki Y, Orlando V, Ovchinnikov DA, Pain A, Passier R, Patrikakis M, Persson H, Piazza S, Prendergast JG, Rackham OJ, Ramilowski JA, Rashid M, Ravasi T, Rizzu P, Roncador M, Roy S, Rye MB, Saijyo E, Sajantila A,Saka A, Sakaguchi S, Sakai M, Sato H, Savvi S, Saxena A, Schneider C, Schultes EA,Schulze-Tanzil GG, Schwegmann A, Sengstag T, Sheng G, Shimoji H, Shimoni Y, Shin JW,Simon C, Sugiyama D, Sugiyama T, Suzuki M, Suzuki N, Swoboda RK, t Hoen PA, Tagami M, Takahashi N, Takai J, Tanaka H, Tatsukawa H, Tatum Z, Thompson M, Toyodo H, Toyoda T, Valen E, van de Wetering M, van den Berg LM, Verado R, Vijayan D, Vorontsov IE,Wasserman WW, Watanabe S, Wells CA, Winteringham LN, Wolvetang E, Wood EJ, Yamaguchi Y, Yamamoto M, Yoneda M, Yonekura Y, Yoshida S, Zabierowski SE, Zhang PG, Zhao X,Zucchelli S, Summers KM, Suzuki H, Daub CO, Kawai J, Heutink P, Hide W, Freeman TC,Lenhard B, Bajic VB, Taylor MS, Makeev VJ, Sandelin A, Hume DA, Carninci P, Hayashizaki Y (2014) A promoter-level mammalian expression atlas. Nature 507, 462–470

[CR8] Cusanovich DA, Hill AJ, Aghamirzaie D, Daza RM, Pliner HA, Berletch JB, Filippova GN, Huang X, Christiansen L, DeWitt WS, Lee C, Regalado SG, Read DF, Steemers FJ, Disteche CM, Trapnell C, Shendure J (2018) A Single-Cell Atlas of. Vivo Mammalian Chromatin Accessibility Cell 174:1309–1324 e1318 30078704 10.1016/j.cell.2018.06.052 PMC6158300

[CR9] Dekker J, Belmont AS, Guttman M, Leshyk VO, Lis JT, Lomvardas S, Mirny LA, O’Shea CC, Park PJ, Ren B, Politz JCR, Shendure J, Zhong S, Network DN (2017) The 4D nucleome project. Nature 549:219–226 28905911 10.1038/nature23884 PMC5617335

[CR10] Dickinson ME, Flenniken AM, Ji X, Teboul L, Wong MD, White JK, Meehan TF, Weninger WJ, Westerberg H, Adissu H, Baker CN, Bower L, Brown JM, Caddle LB, Chiani F, Clary D, Cleak J, Daly MJ, Denegre JM, Doe B, Dolan ME, Edie SM, Fuchs H, Gailus-Durner V, Galli A, Gambadoro A, Gallegos J, Guo S, Horner NR, Hsu CW, Johnson SJ, Kalaga S, Keith LC, Lanoue L, Lawson TN, Lek M, Mark M, Marschall S, Mason J, McElwee ML, Newbigging S, Nutter LM, Peterson KA, Ramirez-Solis R, Rowland DJ, Ryder E, Samocha KE, Seavitt JR, Selloum M, Szoke-Kovacs Z, Tamura M, Trainor AG, Tudose I, Wakana S, Warren J, Wendling O, West DB, Wong L, Yoshiki A, International Mouse Phenotyping C, Jackson L, Infrastructure Nationale Phenomin ICdlS, Charles River, Harwell L, Center MRC, MacArthur RB, Tocchini-Valentini DG, Gao GP, Flicek X, Bradley P, Skarnes A, Justice WC, Parkinson MJ, Moore HE, Wells M, Braun S, Svenson RE, de Angelis KL, Herault MH, Mohun Y, Mallon T, Henkelman AM, Brown RM, Adams SD, Lloyd DJ, McKerlie KC, Beaudet C, Bucan AL, Murray M (2016) SA High-throughput discovery of novel developmental phenotypes. Nature 537, 508–514 10.1038/nature19356 PMC5295821 27626380

[CR11] Gorkin DU, Barozzi I, Zhao Y, Zhang Y, Huang H, Lee AY, Li B, Chiou J, Wildberg A, Ding B, Zhang B, Wang M, Strattan JS, Davidson JM, Qiu Y, Afzal V, Akiyama JA, Plajzer-Frick I, Novak CS, Kato M, Garvin TH, Pham QT, Harrington AN, Mannion BJ, Lee EA, Fukuda-Yuzawa Y, He Y, Preissl S, Chee S, Han JY, Williams BA, Trout D, Amrhein H, Yang H, Cherry JM, Wang W, Gaulton K, Ecker JR, Shen Y, Dickel DE, Visel A, Pennacchio LA, Ren B (2020) An atlas of dynamic chromatin landscapes in mouse fetal development. Nature 583:744–751 32728240 10.1038/s41586-020-2093-3 PMC7398618

[CR12] He W, Sun X, Liu L, Li M, Jin H, Wang WH (2014) The prevalence of chromosomal deletions relating to developmental delay and/or intellectual disability in human euploid blastocysts. PLoS One 9:e85207 24409323 10.1371/journal.pone.0085207 PMC3883698

[CR13] Hinrichs AS, Karolchik D, Baertsch R, Barber GP, Bejerano G, Clawson H, Diekhans M, Furey TS, Harte RA, Hsu F, Hillman-Jackson J, Kuhn RM, Pedersen JS, Pohl A, Raney BJ, Rosenbloom KR, Siepel A, Smith KE, Sugnet CW, Sultan-Qurraie A, Thomas DJ, Trumbower H, Weber RJ, Weirauch M, Zweig AS, Haussler D, Kent WJ (2006) The UCSC Genome Browser Database: update 2006. Nucleic Acids Res 34:D590–598 16381938 10.1093/nar/gkj144 PMC1347506

[CR14] Hirabayashi S, Bhagat S, Matsuki Y, Takegami Y, Uehata T, Kanemaru A, Itoh M, Shirakawa K, Takaori-Kondo A, Takeuchi O, Carninci P, Katayama S, Hayashizaki Y, Kere J, Kawaji H, Murakawa Y (2019) NET-CAGE characterizes the dynamics and topology of human transcribed cis-regulatory elements. Nat Genet 51:1369–1379 31477927 10.1038/s41588-019-0485-9

[CR15] Kaplow IM, Lawler AJ, Schaffer DE, Srinivasan C, Sestili HH, Wirthlin ME, Phan BN, Prasad K, Brown AR, Zhang X, Foley K, Genereux DP, Zoonomia C, Karlsson EK, Lindblad-Toh K, Meyer WK, Pfenning AR (2023) Relating enhancer genetic variation across mammals to complex phenotypes using machine learning. Science 380:eabm7993 37104615 10.1126/science.abm7993 PMC10322212

[CR16] Khurana E, Fu Y, Chakravarty D, Demichelis F, Rubin MA, Gerstein M (2016) Role of non-coding sequence variants in cancer. Nat Rev Genet 17:93–108 26781813 10.1038/nrg.2015.17

[CR17] Kodzius R, Kojima M, Nishiyori H, Nakamura M, Fukuda S, Tagami M, Sasaki D, Imamura K, Kai C, Harbers M, Hayashizaki Y, Carninci P (2006) CAGE: cap analysis of gene expression. Nat Methods 3:211–222 16489339 10.1038/nmeth0306-211

[CR18] Kubo N, Ishii H, Xiong X, Bianco S, Meitinger F, Hu R, Hocker JD, Conte M, Gorkin D, Yu M, Li B, Dixon JR, Hu M, Nicodemi M, Zhao H, Ren B (2021) Promoter-proximal CTCF binding promotes distal enhancer-dependent gene activation. Nat Struct Mol Biol 28:152–161 33398174 10.1038/s41594-020-00539-5 PMC7913465

[CR19] Kubo N, Chen PB, Hu R, Ye Z, Sasaki H, Ren B (2024) H3K4me1 facilitates promoter-enhancer interactions and gene activation during embryonic stem cell differentiation. Mol Cell 84:1742-1752 e1745 38513661 10.1016/j.molcel.2024.02.030 PMC11069443

[CR20] Kvon EZ, Waymack R, Gad M, Wunderlich Z (2021) Enhancer redundancy in development and disease. Nat Rev Genet 22:324–336 33442000 10.1038/s41576-020-00311-x PMC8068586

[CR21] Lloyd KCK, Adams DJ, Baynam G, Beaudet AL, Bosch F, Boycott KM, Braun RE, Caulfield M, Cohn R, Dickinson ME, Dobbie MS, Flenniken AM, Flicek P, Galande S, Gao X, Grobler A, Heaney JD, Herault Y, de Angelis MH, Lupski JR, Lyonnet S, Mallon AM, Mammano F, MacRae CA, McInnes R, McKerlie C, Meehan TF, Murray SA, Nutter LMJ, Obata Y, Parkinson H, Pepper MS, Sedlacek R, Seong JK, Shiroishi T, Smedley D, Tocchini-Valentini G, Valle D, Wang CL, Wells S, White J, Wurst W, Xu Y, Brown SDM (2020) The Deep Genome Project. Genome Biol 21:18 32008577 10.1186/s13059-020-1931-9 PMC6996159

[CR22] Luo Y, Hitz BC, Gabdank I, Hilton JA, Kagda MS, Lam B, Myers Z, Sud P, Jou J, Lin K, Baymuradov UK, Graham K, Litton C, Miyasato SR, Strattan JS, Jolanki O, Lee JW, Tanaka FY, Adenekan P, O’Neill E, Cherry JM (2020) New developments on the Encyclopedia of DNA Elements (ENCODE) data portal. Nucleic Acids Res 48:D882–D889 31713622 10.1093/nar/gkz1062 PMC7061942

[CR23] Lupianez DG, Kraft K, Heinrich V, Krawitz P, Brancati F, Klopocki E, Horn D, Kayserili H, Opitz JM, Laxova R, Santos-Simarro F, Gilbert-Dussardier B, Wittler L, Borschiwer M, Haas SA, Osterwalder M, Franke M, Timmermann B, Hecht J, Spielmann M, Visel A, Mundlos S (2015) Disruptions of topological chromatin domains cause pathogenic rewiring of gene-enhancer interactions. Cell 161:1012–1025 25959774 10.1016/j.cell.2015.04.004 PMC4791538

[CR24] Meehan TF, Conte N, West DB, Jacobsen JO, Mason J, Warren J, Chen CK, Tudose I, Relac M, Matthews P, Karp N, Santos L, Fiegel T, Ring N, Westerberg H, Greenaway S, Sneddon D, Morgan H, Codner GF, Stewart ME, Brown J, Horner N, International Mouse Phenotyping C, Haendel M, Washington N, Mungall CJ, Reynolds CL, Gallegos J, Gailus-Durner V, Sorg T, Pavlovic G, Bower LR, Moore M, Morse I, Gao X, Tocchini-Valentini GP, Obata Y, Cho SY, Seong JK, Seavitt J, Beaudet AL, Dickinson ME, Herault Y, Wurst W, de Angelis MH, Lloyd KCK, Flenniken AM, Nutter LMJ, Newbigging S, McKerlie C, Justice MJ, Murray SA, Svenson KL, Braun RE, White JK, Bradley A, Flicek P, Wells S, Skarnes WC, Adams DJ, Parkinson H, Mallon AM, Brown SDM, Smedley D (2017) Disease model discovery from 3,328 gene knockouts by The International Mouse Phenotyping Consortium. Nat Genet 49:1231–1238 28650483 10.1038/ng.3901 PMC5546242

[CR25] Consortium EP, Moore JE, Purcaro MJ, Pratt HE, Epstein CB, Shoresh N, Adrian J, Kawli T, Davis CA, Dobin A, Kaul R, Halow J, Van Nostrand EL, Freese P, Gorkin DU, Shen Y, He Y, Mackiewicz M, Pauli-Behn F, Williams BA, Mortazavi A, Keller CA, Zhang XO, Elhajjajy SI, Huey J, Dickel DE, Snetkova V, Wei X, Wang X, Rivera-Mulia JC, Rozowsky J, Zhang J, Chhetri SB, Zhang J, Victorsen A, White KP, Visel A, Yeo GW, Burge CB, Lecuyer E, Gilbert DM, Dekker J, Rinn J, Mendenhall EM, Ecker JR, Kellis M, Klein RJ, Noble WS, Kundaje A, Guigo R, Farnham PJ, Cherry JM, Myers RM, Ren B, Graveley BR, Gerstein MB, Pennacchio LA, Snyder MP, Bernstein BE, Wold B, Hardison RC, Gingeras TR, Stamatoyannopoulos JA, Weng Z (2020) Expanded encyclopaedias of DNA elements in the human and mouse genomes. Nature 583:699–710 32728249 10.1038/s41586-020-2493-4 PMC7410828

[CR26] Nobusada T, Yip CW, Agrawal S, Severin J, Abugessaisa I, Hasegawa A, Hon CC, Ide S, Koido M, Kondo A, Masuya H, Oki S, Tagami M, Takada T, Terao C, Thalhath N, Walker S, Yasuzawa K, Shin JW, de Hoon MJL, Carninci P, Kawaji H, Kasukawa T (2025) Update of the FANTOM web resource: enhancement for studying noncoding genomes. Nucleic Acids Res 53:D419–D424 39592010 10.1093/nar/gkae1047 PMC11701582

[CR27] Oguchi A, Suzuki A, Komatsu S, Yoshitomi H, Bhagat S, Son R, Bonnal RJP, Kojima S, Koido M, Takeuchi K, Myouzen K, Inoue G, Hirai T, Sano H, Takegami Y, Kanemaru A, Yamaguchi I, Ishikawa Y, Tanaka N, Hirabayashi S, Konishi R, Sekito S, Inoue T, Kere J, Takeda S, Takaori-Kondo A, Endo I, Kawaoka S, Kawaji H, Ishigaki K, Ueno H, Hayashizaki Y, Pagani M, Carninci P, Yanagita M, dagger, Parrish IC, Terao N, Yamamoto C, Murakawa K, Members Y (2024) IC An atlas of transcribed enhancers across helper T cell diversity for decoding human diseases. Science 385, eadd8394 10.1126/science.add8394 38963856

[CR28] Oki S, Ohta T, Shioi G, Hatanaka H, Ogasawara O, Okuda Y, Kawaji H, Nakaki R, Sese J, Meno C (2018) ChIP-Atlas: a data-mining suite powered by full integration of public ChIP-seq data. EMBO Rep 19 10.15252/embr.201846255 PMC6280645 30413482

[CR29] Osterwalder M, Barozzi I, Tissieres V, Fukuda-Yuzawa Y, Mannion BJ, Afzal SY, Lee EA, Zhu Y, Plajzer-Frick I, Pickle CS, Kato M, Garvin TH, Pham QT, Harrington AN, Akiyama JA, Afzal V, Lopez-Rios J, Dickel DE, Visel A, Pennacchio LA (2018) Enhancer redundancy provides phenotypic robustness in mammalian development. Nature 554:239–243 29420474 10.1038/nature25461 PMC5808607

[CR30] Panigrahi A, O’Malley BW (2021) Mechanisms of enhancer action: the known and the unknown. Genome Biol 22:108 33858480 10.1186/s13059-021-02322-1 PMC8051032

[CR31] Perenthaler E, Yousefi S, Niggl E, Barakat TS (2019) Beyond the exome: the non-coding genome and enhancers in neurodevelopmental disorders and malformations of cortical development. Front Cell Neurosci 13:352 31417368 10.3389/fncel.2019.00352 PMC6685065

[CR32] Reyna J, Fetter K, Ignacio R, Marandi CCA, Ma A, Rao N, Jiang Z, Figueroa DS, Bhattacharyya S, Ay F (2025) Loop catalog: a comprehensive HiChIP database of human and mouse samples. Genome Biol 26:175 40542429 10.1186/s13059-025-03615-5 PMC12180236

[CR33] Rheinbay E, Nielsen MM, Abascal F, Wala JA, Shapira O, Tiao G, Hornshoj H, Hess JM, Juul RI, Lin Z, Feuerbach L, Sabarinathan R, Madsen T, Kim J, Mularoni L, Shuai S, Lanzos A, Herrmann C, Maruvka YE, Shen C, Amin SB, Bandopadhayay P, Bertl J, Boroevich KA, Busanovich J, Carlevaro-Fita J, Chakravarty D, Chan CWY, Craft D, Dhingra P, Diamanti K, Fonseca NA, Gonzalez-Perez A, Guo Q, Hamilton MP, Haradhvala NJ, Hong C, Isaev K, Johnson TA, Juul M, Kahles A, Kahraman A, Kim Y, Komorowski J, Kumar K, Kumar S, Lee D, Lehmann KV, Li Y, Liu EM, Lochovsky L, Park K, Pich O, Roberts ND, Saksena G, Schumacher SE, Sidiropoulos N, Sieverling L, Sinnott-Armstrong N, Stewart C, Tamborero D, Tubio JMC, Umer HM, Uuskula-Reimand L, Wadelius C, Wadi L, Yao X, Zhang CZ, Zhang J, Haber JE, Hobolth A, Imielinski M, Kellis M, Lawrence MS, von Mering C, Nakagawa H, Raphael BJ, Rubin MA, Sander C, Stein LD, Stuart JM, Tsunoda T, Wheeler DA, Johnson R, Reimand J, Gerstein M, Khurana E, Campbell PJ, Lopez-Bigas N, Drivers P, Functional Interpretation Working G, Group PSVW, Weischenfeldt J, Beroukhim R, Martincorena I, Pedersen JS, Getz G, Consortium P (2020) Analyses of non-coding somatic drivers in 2,658 cancer whole genomes. Nature 578, 102–111

[CR34] Schaeffer L, Lindner L, Pavlovic G, Herault Y, Birling MC (2023) CRISMERE chromosome engineering in mouse and rat. Methods Mol Biol 2631:277–297 36995673 10.1007/978-1-0716-2990-1_12

[CR35] Schmitt AD, Hu M, Jung I, Xu Z, Qiu Y, Tan CL, Li Y, Lin S, Lin Y, Barr CL, Ren B (2016) A compendium of chromatin contact maps reveals spatially active regions in the human genome. Cell Rep 17:2042–2059 27851967 10.1016/j.celrep.2016.10.061 PMC5478386

[CR36] Sokhi UK, Liber MP, Frye L, Park S, Kang K, Pannellini T, Zhao B, Norinsky R, Ivashkiv LB, Gong S (2018) Dissection and function of autoimmunity-associated TNFAIP3 (A20) gene enhancers in humanized mouse models. Nat Commun 9:658 29440643 10.1038/s41467-018-03081-7 PMC5811492

[CR37] Wilson R, Bulbul Atac T, Cheng TK, Frost A, Gunes O, Kan M, Keskivali-Bond P, Lopez Gomez F, McLaughlin J, Mucha J, Munava T, Oliveira C, Pava D, Pena Estrada JF, Selkirk E, Vardal B, Wells S, Cacheiro P, Smedley D, Parkinson H (2026) International mouse phenotyping consortium portal: facilitating investigation of gene function and providing insights into human disease. Nucleic Acids Res 54:D1133–D1142 41231752 10.1093/nar/gkaf1148 PMC12807668

[CR38] Zhang K, Hocker JD, Miller M, Hou X, Chiou J, Poirion OB, Qiu Y, Li YE, Gaulton KJ, Wang A, Preissl S, Ren B (2021) A single-cell atlas of chromatin accessibility in the human genome. Cell 184:5985–6001 34774128 10.1016/j.cell.2021.10.024 PMC8664161

